# Prevalence of depressive disorders among pregnant women in Brazil in 2019: A descriptive cross-sectional study

**DOI:** 10.1590/1516-3180.2023.0238.R1.03072024

**Published:** 2024-12-20

**Authors:** Maira Gonçalves de Oliveira Lucas, Maria Isabel do Nascimento

**Affiliations:** I Assistant Professor, Escola de Medicina Souza Marques, Fundação Técnico Educacional Souza Marques, Rio de Janeiro (RJ), Brazil.; II Adjunct Professor, Department of General and Specialized Surgery, Faculty of Medicine, Universidade Federal Fluminense (UFF), Niterói (RJ), Brazil.

**Keywords:** Depressive disorder., Pregnancy., Patient health questionnaire., Prevalence., Cross-sectional studies., Anhedonia., Mental health in pregnancy., Diagnosis of depression., Suicidal thoughts., National Health Surveys., Perinatal depression.

## Abstract

**BACKGROUND:**

Prenatal depression threatens maternal and child well-being and interferes with issues prioritized by the 2030 agenda for the Sustainable Development Goals.

**OBJECTIVES:**

This study aimed to estimate the prevalence of depressive disorders during pregnancy using the Patient Health Questionnaire-9 (PHQ-9).

**DESIGN AND SETTING:**

A cross-sectional study using a sample of pregnant Brazilian women, representative of Brazil as a whole.

**METHODS:**

Data were obtained from the National Health Survey 2019 (PNS-2019), as coordinated by the Instituto Brasileiro de Geografia e Estatística (IBGE). Women aged 18-49 years who were pregnant during PNS-2019 data collection were included. The prevalence of depressive disorders and 95% confidence intervals (95%CI) were calculated.

**RESULTS:**

The prevalence of depression before the current pregnancy was 6.03% (95%CI: 3.80%; 8.25%). Using the PHQ-9, the prevalence of Major Depressive Disorder (MDD) in the current pregnancy was estimated to be 17.39% (95%CI: 12.70%–22.06%) among pregnant women who were targeted by the PNS-2019. Moreover, MDD was 15.26% (95%CI: 10.54%; 19.97%) among those who were free from depression history and in half of the pregnant woman population with depression history. Suicidal ideation has been reported in almost 23% of pregnant women with a history of depression. The two items from Patient Health Questionnaire-2 (PHQ-2) (anhedonia and depressed mood) with a cutoff of ≥ 3 presented similar results.

**CONCLUSIONS:**

Depression during pregnancy affects a non-negligible proportion of pregnant women, thus constituting an important public health problem. Therefore, it is crucial to discuss the implementation of regular screening for depression during prenatal care programs in Brazil.

## INTRODUCTION

The 2030 agenda for the Sustainable Development Goals established the following as its 3rd objective: “to ensure a healthy life and promote well-being for all at all ages”. Linked to this objective is a list of subheadings directly related to maternal and child health, including item 3.4, which is aligned with promoting mental health and well-being.^1^ Throughout the life cycle, well-being and mental health are threatened by risk factors shared by the mother and child in various developmental phases: preconception, perinatal and postnatal, adolescence, and youth.^2^

During the gestational period, women experience physical, psychological, and emotional changes as permeated by stressful situations, few sleep hours, hormonal oscillations, and tiredness, thereby making it difficult to suspect and diagnose depression.^3^ Analysis of the current nationwide Brazilian data indicates that the underdiagnosis of depression is more frequent among pregnant women (88.1%), as compared to non-pregnant women (68.0%).^4^ Women of reproductive age are common users of health services, especially during pregnancy, a scenario that provides an opportunity to track depressive symptoms and make referrals to specialized professionals.^4^

When screening for depressive disorders, the American Psychiatric Association (APA) recommends investigating the frequency of nine symptoms, provided that they differ from the previously perceived functioning, occur nearly every day, and persist over the previous two-week period.^5^ The investigated symptoms should correspond to the items in the Patient Health Questionnaire-9 (PHQ-9), a tool proposed to diagnose mental disorders among primary healthcare users.^6^ The PHQ-9 is an instrument validated across different scenarios and is used in people with different health conditions and age characteristics.^7^

The PHQ-9 has proven to be reliable in tracking suspected cases of Major Depressive Disorders (MDD) among the general population in Brazil,^8^ and has revealed acceptable reliability and validity for screening depressive symptoms among pregnant women.^9^ It is included as one of the modules of the National Health Survey, a population-based survey representing the Brazilian population.^10^ It provides population-based information and measures the frequency of prenatal depression, which interferes with several issues prioritized by the Sustainable Development Goals, and can contribute to the planning and implementation of actions aimed at achieving the 2030 Agenda.

## OBJECTIVE

This study aimed to estimate the prevalence of depressive disorders among pregnant women in Brazil by using data from the National Health Survey (PNS-2019).

## METHODS

This descriptive study addressed the research question, “what is the prevalence of depression during pregnancy in Brazil?”. The data was obtained from the National Health Survey-2019 (PNS-2019).^10^ The PNS has the merit of providing public access to information regarding the health conditions of populations residing in private households within the national territory, which is not made available by the country’s information systems.^11^ The original project of the PNS-2019 was approved by the National Commission for Ethics in Research (CONEP) under the National Health Council (CNS), with the approval number: No. 3.529.376, dated 23^rd^ August 2019.

### Access to the 2019 PNS microdata

The microdata was imported using the online option via the “PNSIBGE” package and the R program, as recommended by IBGE.^12^ The microdata was derived from the “questionnaire of the selected resident”, which gathered the questions asked only to the randomly selected residents in the selected household. The steps were followed in order to facilitate the analysis via the “survey” package.

### Sampling plan

Details of the sampling plan can be retrieved from the article, “National Health Survey 2019: history, methods and perspectives”.^11^ In summary, the method considered the effects of the selection scheme applied in the complex sampling plan, as well as the measures of error of the estimates and the originally applied post-stratification estimator.^12^

### Participants

The target population of the PNS-2019 included people aged ≥ 15 years who lived in permanent private households in rural or urban areas. This allowed for the disaggregation of (i) macro-regions, (ii) federative units (UF), (iii) Capitals and (iv) Metropolitan Regions. This study used PNS-2019 data to produce estimates for the target population of women aged 18–49 years. The subset included women with a confirmed pregnancy during PNS data collection.

### Variables of interest selected from the PNS-2019

a) Module: general characteristics: age in years (18-24, 25-34, and 35-49); ethnicity/race (white, black, yellow, brown, and indigenous); having a spouse or partner living in the same household (yes versus no).
b) Module: lifestyle: pregnancy status (yes versus no) at the time of the interview [Question P005. Are you currently pregnant?]
c) Module: chronic diseases: History of a medical diagnosis of depression [Question Q092. Had a doctor or mental health professional [such as a psychiatrist or psychologist] ever diagnosed you with depression?
d) Module: perception of health status: symptoms within the last two weeks that could have affected the respondent in a different way than usual. Symptoms were assessed using the PHQ-9, which is a self-reporting instrument composed of nine items with four response options as follows: 0 (none of the days), 1 (less than half of the days), 2 (more than half of the days), and 3 (almost every day).^6^ The PNS-2019 applied the PHQ-9 items in the following order: N010 (trouble falling or staying asleep, or sleeping too much), N011 (feeling tired or having little energy), N012 (little interest or pleasure in doing things), N013 (trouble concentrating on things), N014 (poor appetite or overeating), N015 (moving or speaking so slowly, or the opposite), N016 (feeling down, depressed, or hopeless), N017 (feeling bad about yourself, or that you are a failure or have let yourself or your family down), and N018 (thoughts that you would be better off dead or of hurting yourself).

### Definition and measurement of depressive manifestations

Depressive manifestations were measured as follows:

(i) Frequency of a history of depression, as diagnosed by a healthcare professional according to question Q092 (Had a doctor or mental health professional [such as a psychiatrist or psychologist] ever diagnosed you with depression?). The answer options were “yes” versus “no”, indicating positive or negative previous depression before the current pregnancy.
(ii) Frequency of depressive symptoms in the last 15 days according to the responses to the PHQ-9,^6^ considering:
  a) Firstly, the PHQ-9 was interpreted as a continuous scale in which the sum varied from 0 to 27; a cutoff point of ≥ 10 suggested MDD.
  b) Subsequently, we scrutinized two questions (“lack of pleasure in doing things” and “depressed mood”) comprising the PHQ-2.^13^ The PHQ-2 interpretation was based on summed scores ranging from 0 to 6; a cutoff point of ≥ 3 suggested depression disorders.^13^ In our study, the N12 (lack of pleasure in doing things) and N16 (depressed mood) items were used to check the distribution of pregnant women in the different cutoff points and to obtain the estimates of prevalence affecting those with a cutoff point of ≥ 3, which were suspected with depression disorders.

The frequency of depression was calculated considering the following participants of the PNS-2019:

1) All pregnant women as a whole;
2) The subgroup of pregnant women restringing those with previous depression (Question Q092 with a positive response);
3) The subgroup of pregnant women restringing those without previous depression (Question Q092 with a negative response);

This allowed for the calculation of the prevalence of depressive disorders that manifested during the gestational period of: (i) pregnant women as a whole, (ii) pregnant women with a previous medical diagnosis of depression, and (iii) pregnant women without a history of depression.

### Statistical analysis

Data analysis was performed online according to the recommendations of the IBGE for complex samples.^12^ The Survey package was used; the function, design = subset, and the argument, selected = TRUE, were performed. This was followed by the calculation of prevalence and 95%CIs.

## RESULTS

The data analyzed in this study were representative of a contingent of women expected to be pregnant (n = 1,402,399) at the time of data collection (from 2019-August to 2019-December 2019) of the National Health Survey in Brazil. The results indicated a prevalence of 6.03% (95%CI: 3.80%; 8.25%) for a previously self-reported medical diagnosis of depression. Most pregnant women reported living with a partner at home, regardless of a history of depression. **Table 1** summarizes the demographic characteristics of the pregnant women analyzed in this study.

**Table 1:** Demographic characteristics of the target population of women aged 18 to 49 years who were pregnant at the time of the National Health Survey (PNS-2019) data collection, Brazil, 2019

	Total (n = 1,402,399)		With a previous depression diagnosis (n = 84,569)		Without a previous depression diagnosis (n = 1,317,830)	
	Prevalence	95%CI	Prevalence	95%CI	Prevalence	95%CI
**Age**						
18 a 24	34.6	29.1; 40.2	17.4	4.2; 30.6	35.7	29.9; 41.5
25 a 34	42.5	36.7; 48.3	36.9	18.2; 55.7	42.8	36.7;48.8
35 a 49	22.9	17.5; 28.3	45.6	27.2; 63.9	21.4	15.8; 27.0
**Skin Color**						
White	33.3	27.6; 39.0	54.4	36.2;72.6	31.9	26.0; 37.8
Black	15.9	11.2; 20.5	9.8	-15.1; 21.2	16.3	11.3; 21.1
Yellow	0.5	-0.3;1.4	0.0	0.0; 0.0	0.5	-0.3; 1.4
Mixed	49.9	43.7; 56.1	35.7	19.1; 52.3	50.8	44.2; 57.2
Indigenous	0.4	-0.02; 0.9	0	0.0; 0.0	0.5	0.0; 0.9
**Living with partner**						
Yes	78.7	73.8; 83.6	73.9	56.1; 91.7	79.1	73.9; 84.1
No	21.3	16.4; 26.1	26.1	8.2; 4.4	20.9	15.8; 26.0

Data source: National Health Survey, 2019. Instituto Brasileiro de Geografia e Estatística. CI = confidence interval.

### Prevalence of depression symptoms via PHQ-9 items

The symptoms captured by each PHQ-9 item indicated that suicidal ideation had the lowest positivity. The will to die was confirmed in < 5% of pregnant women as a whole (4.47%) and of those without a history of depression (3.31%). However, 22.51% of participants with a history of depression reported suicidal thoughts (**Table 2**).

**Table 2:** Frequency of depressive symptoms, as measured by the Patient Health Questionnaire-9 administered to pregnant women participants in the National Health Survey (PNS-2019), in Brazil, 2019

Pregnant women group/PHQ-9-symptoms	None of the days	Less than half of the days	More than half of the days	Almost everyday
	(%)	(%)	(%)	(%)
**All pregnant women together**				
N010 (sleep problems)	55.56	20.72	8.89	14.83
N011 (low energy)	47.29	25.75	12.79	14.16
N012 (anhedonia)	55.18	27.27	7.59	9.95
N013 (concentration difficulties)	75.97	13.92	5.50	4.59
N014 (appetite changes)	67.61	15.63	6.75	9.99
N015 (moving or speaking so slowly)	77.67	12.73	4.29	5.28
N016 (depressed mood)	67.52	22.19	5.15	5.13
N017 (feeling bad about yourself)	84.45	10.63	1.42	3.48
N018 (suicidal thoughts)	95.53	3.08	0.61	0.76
**Pregnant women with a previous depression diagnosis**				
N010 (sleep problems)	18.65	21.13	10.69	49.53
N011 (low energy)	23.89	26.35	11.34	38.42
N012 (anhedonia)	35.31	30.54	13.32	20.83
N013 (concentration difficulties)	51.38	15.26	7.93	25.43
N014 (appetite changes)	48.17	18.03	9.17	24.63
N015 (moving or speaking so slowly)	52.23	18.11	5.48	24.18
N016 (depressed mood)	43.78	23.83	8.45	23.93
N017 (feeling bad about yourself)	56.74	18.88	11.23	13.15
N018 (suicidal thoughts)	77.49	9.47	9.52	3.52
**Pregnant women without a previous depression diagnosis**				
N010 (sleep problems)	57.92	20.70	8.77	12.61
N011 (low energy)	48.79	25.72	12.89	12.60
N012 (anhedonia)	56.45	27.06	7.23	9.26
N013 (concentration difficulties)	77.55	13.84	5.35	3.26
N014 (appetite changes)	68.87	15.48	6.60	9.05
N015 (moving or speaking so slowly)	79.31	12.39	4.22	4.07
N016 (depressed mood)	69.05	22.08	4.94	3.92
N017 (feeling bad about yourself)	86.23	10.10	0.80	2.87
N018 (suicidal thoughts)	96.69	2.67	0.04	0.59

Data Source: National Health Survey 2019. Instituto Brasileiro de Geografia e Estatística. 95% Confidence Intervals were omitted.

### Prevalence of MDD by a cutoff point of ≥ 10

Using the PHQ-9 items as a continuous scale, the mean total score for pregnant women was 4.62 (95%CI: 3.98; 5.26). The lowest and highest means were 4.26 (95%CI: 3.63; 4.89) for the pregnant women subgroup without a history of depression and 10.26 (95% CI: 7.47; 13.05) for those with a history of depression, respectively. The estimates based on a cutoff point of ≥ 10 suggested that MDD could be present in approximately 17.39% (95%CI: 12.70%; 22.06%) of pregnant women who were targeted by the PNS-2019. The prevalence of MDD was 15.26% (95%CI: 10.54%; 19.97%) among those who were free from previous depression and among approximately half (95%CI: 31.98%; 69.01%) of pregnant women with previous depression.

### Depressive disorder as measured by PHQ-2 [symptoms of anhedonia (N012) and depressed mood (N016)]

The mean scores of the two PHQ-2 items was 1.20 (95%CI: 1.01; 1.39), 2.32 (95%CI: 1.37; 3.26), and 1.13 (95%CI: 0.94; 1.31), among pregnant women as a whole, those with previous depression, and those without depression, respectively. The positivity for depressive disorders according to different cutoff points indicated that the higher the cutoff point, the lower the prevalence of depression. Moreover, estimates with a cutoff point of ≥ 3 suggested that 16.61% (all pregnant women), 32.22% (group with depression history), and 15.61% (group without depression history) of pregnant women in Brazil may have positive depressive symptoms (**Table 3**).

**Table 3:** Positivity of Patient Health Questionnaire-2, considering two-item scores (N012-anhedonia and N016-depressed mood) and different cutoff points among pregnant women with and without previous depression

Pregnant women/ Cutoff points	Positivity (n)	Prevalence (%)	95%CI
**All together (n = 1,402,399)**			
≥ 1	739,347	52.72	46.92; 58.51
≥ 2	465,544	33.19	27.46; 38.92
≥ **3**	**232,964**	**16.61**	**12.02; 21.20**
≥ 4	138,840	9.90	5.89; 13.90
≥ 5	64,981	4.63	2.31; 6.95
**With a previous depression diagnosis (n = 84,569)**			
≥ 1	60,889	72.00	56.11; 87.88
≥ 2	45,050	53.27	34.76; 71.77
≥**3**	**27,255**	**32.22**	**13.50; 50.95**
≥ 4	26,046	30.79	12.04; 49.55
≥ 5	19,519	23.08	4.87; 41.28
**Without a previous depression diagnosis (n = 1,317,8300)**			
≥ 1	678,457	51.48	45.42; 57.54
≥ 2	420,493	31.90	25.94; 37.87
≥**3**	**205,709**	**15.61**	**10.91; 20.30**
≥ 4	112,794	8.55	4.52; 12.58
≥ 5	45,461	3.44	1.38; 5.51

Data Source: National Health Survey, 2019. Instituto Brasileiro de Geografia e Estatística; CI = confidence intervals.

The overlapping of the 95%CIs of the estimates captured via the PHQ-9 items (dichotomized at a cutoff point of ≥ 10) as compared to those from the PHQ-2 items (dichotomized at a cutoff point of ≥ 3), which are displayed in **Table 3**, suggested no difference between the two methods in terms of capturing depressive disorders during pregnancy, regardless of previous depression, among Brazilian women.

### DISCUSSION

This study focused on the mental health of pregnant women and showed that symptoms suggestive of depression during pregnancy may be present in > 15% of the population. The estimates derived from the PHQ-9 indicated that among pregnant women, MDD may be present in 17.39% (pregnant women as a whole), 15.26% (pregnant women, without previous depression), and 50.00% (pregnant women, with previous depression) of the subgroups analyzed. The two PHQ-2 items with a cutoff point of ≥ 3 showed similar results.

Significant public investment in Brazil has been made to improve obstetric and perinatal outcomes. Despite the harmful effects caused by maternal depression on various aspects of women’s and children’s health and family relationships, it remains underdiagnosed and inadequately studied in Brazil.^14^ To reduce the literature gap in the context of national representation, the hospital-based survey, “Nascer no Brasil”, estimated a post-partum depression frequency of 26.3%, based on information collected through telephone calls between 6 and 18 months after child birth.^15^ The collected information led to the creation of a risk model for post-partum depression, wherein the predictor, “previous history of mental disorders”, constituted the strongest risk factor after final model adjustments.^15^

In line with the aforementioned results, the present study detected a prevalence of 6.05% for depression, as self-reported by PNS-2019 participants who were diagnosed before pregnancy. Moreover, data from 6814 mother-child pairs, which were analyzed to investigate associations between maternal mental health disorders before and during pregnancy, showed that depression increased the risk of anxiety disorders during pregnancy and pediatric healthcare utilization.^16^ In addition to these issues, the present study detected a high frequency of symptoms, thereby indicating that MDD affects pregnant women with (50.0%) and without (15.2%) a history of depression. These figures suggest a need to implement depression-screening measures, regardless of mental health status before pregnancy.

Therefore, the PHQ-2 appears to be an effective and sensitive alternative when screening for depressive disorders. This instrument comprises two core PHQ-9 items and has been recommended for use in high demand scenarios, wherein healthcare professionals are overloaded and have little time to carry out everyday tasks.^13^ A study conducted in the United States using the PHQ-2 on a sample size of 218 pregnant women with a gestational age of up to 16 weeks showed sensitivity and specificity measures of 77% and 59%, respectively, with a cutoff point of ≥ 3 and the Composite International Diagnostic Interview as reference measures.^17^ In Spain, the performance of the PHQ-2 was evaluated in a study that included 1019 pregnant women in their first trimester.^18^ The instrument showed sensitivity and specificity measures of 85.4% and 79.5%, respectively, when utilizing a cutoff point of ≥ 2 and the PHQ-9 as the gold standard, with excellent discriminatory ability (84%).^18^

In addition to the long list of implications attributed to perinatal depression,^19,20^ it is crucial to draw attention to the fact that almost 5.0% and 23.0% of pregnant Brazilian women, as a whole and among those with previous depression, respectively, may be living with suicidal thoughts and are at risk for non-fatal and fatal outcomes. This result was extracted from a PHQ-9 component item (N018-suicidal thoughts) that was analyzed in isolation. This item was also analyzed in a study on pregnant women in Spain, which showed the occurrence of suicidal ideation in 2.6% of those interviewed in their first trimester.^21^

Suicidal thoughts may manifest from the beginning of the gestational period and may extend into the post-partum period. Results from a study with a sample of 831 low-income Brazilian women who were pregnant between the 20^th^ and 30^th^ weeks estimated a 6.3% prevalence of antenatal suicidal ideation.^22^ Pooled measures from a meta-analysis that included 71 studies showed that the prevalence of suicidal ideation was 10% during the gestational period, 7% in the post-partum period, and 8% in both groups. In addition, the studies reported a higher frequency (13%) in low- and middle-income countries, including five studies conducted in Brazil.^23^

Although rarely practiced, suicide attempts were also measured by a meta-analysis that gathered 14 studies and indicated that the act has been detected during pregnancy (prevalence 95%CI: 0.10%; 4.69%) and the first post-partum year (prevalence 95%CI: 0.01%; 3.21%).^24^ A retrospective cohort study conducted with data from 712 hospitals in Japan included 1202 pregnant women admitted during the pre-partum period and 111 re-admissions within 1-year post-partum, of which all admissions were due to suicide attempts.^25^ In addition, prenatal psychiatric disorders, including depression, were among the potential risk factors for peripartum suicide attempts.^25^ Suicide attempts among women in reproductive age are increasing in Brazil.^26^ However, the magnitude of these events practiced during the gestational period demands further studies for better understanding and urgent proposal of intervention measures.

The present study used data from a representative sample of women residing in Brazil to provide measures on the prevalence of depressive disorders during pregnancy. However, this study has some limitations. First, the data analyzed corresponded to a subset of selected residents derived from a complex sampling process that included age classes in the definition of expansion factors,^10^ whose limits were not fully equivalent to the age group of the study. Nevertheless, we believe that the estimated parameters facilitate population inferences for women aged 18–49 years who were pregnant according to the PNS-2019. Another issue that must be pointed out relates to the investigation of symptoms that are commonly present in some general medical conditions, as well as in pregnancy.^5^ In general, gestation is physiologically characterized by changes in appetite and sleep, which are also investigated by two PHQ-9 items. Bearing in mind the equivalence of the measures provided by the different structures involving appetite and sleep investigation, it is possible that such a limitation may have been circumvented to some extent. Finally, it is important to point out that despite the practicality of using the PHQ-2 to identify people suspected of suffering from depressive disorders, it is only a screening instrument that indicates whether the person needs evaluation using more appropriate instruments and/or specialized professionals. Despite the adequacy demonstrated in capturing cases of depression among women,^27^ the performance of the instrument still requires further evaluation among pregnant women. Checking of validity and reliability of PHQ-9 and PHQ-2 in Brazilian pregnant women deserves consideration in future research.

## CONCLUSION

In conclusion, the present study shows that the symptoms of suicidal ideation in isolation and MDD are highly prevalent conditions during pregnancy, thus supporting the formulation of guidelines for regular screening during prenatal care. The equivalence of the estimates, as measured by the PHQ-9 items interpreted in two different ways, suggests that the two questions about depressive mood and anhedonia could constitute a screening tool for gestational depression, as they allow for rapid administration and are suitable for use in high-demand environments, such as primary care in Brazil.

The results of this research were presented as an E-poster titled, “Prevalência de transtornos depressivos na gestação de acordo com a Pesquisa Nacional de Saúde – 2019”, at the 27° Congresso Paulista de Obstetrícia e Ginecologia, 11-13 de agosto de 2022 – Transamerica Expo Center – São Paulo (SP)
